# Quality of life and Alzheimer’s disease: Influence of participation in a rehabilitation center

**DOI:** 10.1590/S1980-57642009DN30300011

**Published:** 2009

**Authors:** Fernanda Machado, Paula V. Nunes, Luciane F. Viola, Franklin S. Santos, Orestes V. Forlenza, Mônica S. Yassuda

**Affiliations:** 1 B.S., Gerontology, Day Hospital Rehabilitation Center (CRHD), Department and Institute of Psychiatry (IPq), Faculty of Medicine (FMUSP), University of São Paulo, São Paulo, SP, Brazil; 2 M.D., PhD in Psychiatry, University of São Paulo, São Paulo, SP, Brazil; 3 Psychologist; 4 M.D., Geriatrics, Joint Professor and PhD, Department of Clinical Medicine, University of Sao Paulo, São Paulo, SP, Brazil; 5 M.D., PhD in Psychiatry, University of São Paulo, São Paulo, SP, Brazil; 6 PhD in Developmental Psychology; Assistant Professor of Gerontology, School of Arts, Sciences and Humanities, University of São Paulo, São Paulo, SP, Brazil.

**Keywords:** Alzheimer’s disease, quality of life, psychosocial intervention

## Abstract

Quality of life is seldom explored in evaluations of therapeutic interventions in Alzheimer’s disease.

**Objective:**

To verify whether participation in a cognitive and functional rehabilitation program improves quality of life (QOL) among Alzheimer’s disease (AD) patients.

**Methods:**

19 AD patients participated in this study, 12 of whom attended 24 multi-professional intervention sessions – the experimental group – whereas the remaining 7 comprised the control group. The following tools were used to assess changes: a) Mini-Mental State Examination (MMSE); b) Geriatric Depression Scale (GDS); c) Quality of Life in AD evaluation scale (QOL-AD); d) Open question on QOL.

**Results:**

Participation had no positive impact on quantitative clinical variables (MMSE, GDS, QOL-AD). The answers to the open question, examined using the Collective Subject Discourse (CSD) method, suggested that QOL improved after the intervention.

**Conclusion:**

Combining pharmacological treatment with psychosocial intervention may prove to be an effective strategy to enhance the QOL of AD patients.

## Abstract (pt)

Qualidade de vida não é frequentemente explorada na avaliação dos resultados de intervenções terapêuticas em DA.

**Objetivo:**

Verificar se a participação em um programa de reabilitação cognitiva e funcional melhora a qualidade de vida (QV) de pacientes com doença de Alzheimer.

**Metodos:**

19 pacientes com DA participaram deste estudo, 12 participaram de 24 sessões de intervenção multiprofissional – grupo experimental – enquanto os 7 restantes compuseram o grupo controle. Os seguintes instrumentos foram utilizados para avaliar mudanças: a) Mini-Exame do Estado Mental (MEEM); b) Escala de Depressão Geriátrica (EDG); c) Escala de Avaliação de Qualidade de Vida na Doença de Alzheimer (QdV-DA); d) questão aberta sobre QV.

**Resultados:**

A participação não mostrou efeitos positivos em variáveis clínicas quantitativas (MEEM, EDG, QdV-DA). Os relatos na questão aberta, analisados através da metodologia do discurso do sujeito coletivo (DSC), sugeriram que a QV melhora após a intervenção.

**Conclusão:**

Aliar o tratamento medicamentoso às intervenções psicossociais pode ser uma boa estratégia para a melhora da QV dos portadores de DA.

Great advances have been made since the first characterization of Alzheimer’s disease (AD) in 1906, ranging initially from the elucidation of the biochemical stages leading to neuronal death, to modern day pharmacological treatment and psychosocial intervention strategies aimed at AD patients as well as their caregivers and family. In spite of these advances, a cure or therapy able to halt the progress of the disease has yet to be found. Therefore, symptom relief and management, as well as psychosocial support, still constitute the best alternative for patients and their family.

Psychosocial interventions (PIs) for patients suffering from AD can be defined as a concerted set of biomedical, psychological, social and educational interventions that contribute toward enhancing or maintaining the state of health and quality of life of patients and their caregivers. PIs are aimed at complementing pharmacological treatment of AD and are being increasingly employed for this purpose. There is a wide range of potential applications of PIs including: neuropsychological rehabilitation, cognitive training, environmental changes, nutritional orientation, physical activity, and psychological counseling and support for family members and caregivers. However, few studies are available which assess the benefits derived from PIs,^1^ in spite of the importance of PIs in as far as they take into account not only the biological aspects of the person but also the individual as a whole. PIs have the potential to make a significant impact on patients’ and caregivers’ quality of life.

Quality of Life (QOL) is a topic that has been widely discussed and investigated recently across numerous fields of knowledge. The World Health Organization Quality of Life Group (WHOQOL Group) has become a reference concerning health-related Quality of Life, defining it as “individuals’ perceptions of their position in life in the context of the culture and value systems in which they live and in relation to their goals, expectations, standards and concerns.”^2^ In dementia, the concept of QOL must encompass the integration of cognitive functioning, daily-life activities, social interaction and psychological well-being.^3^ Consequently, the QOL variable can be included as a measure of effectiveness of therapeutic interventions in AD, along with conventionally used parameters.

The objective of the present study was to determine whether participation of AD patients at a multidisciplinary rehabilitation center influences their quality of life.

## Methods

### Materials and methods

This research work was carried out at the Day Hospital Rehabilitation Center (CRHD) of the Department and Institute of Psychiatry (IPq), Faculty of Medicine (FMUSP), University of Sao Paulo. Both quantitative and qualitative methods were used as efficacy measures in this research. Pre and post-intervention interviews were conducted by a researcher who was blinded to patient assignment (experimental or control). These interviews focused on patients’ quality of life. The intervention comprised 24 sessions and was carried out between March and July 2008, upon approval by the local Ethics Committee.

For this study, 19 individuals aged 65 and older, both men and women, diagnosed with mild to moderate AD were selected to participate. These individuals were assigned to one of two groups: experimental or control.

The criteria for inclusion in the research protocol were: AD diagnosis according to parameters adopted by the National Institute for Communicative Disorders and Stroke – Alzheimer’s Disease and Related Disorders Association – NINCDS-ADRA;^4^ regular use of specific AD pharmacological treatment for at least three months at the same daily dosage; mild, sometimes moderate stage of AD; availability to participate in the intervention during a 12-week period; agreement to take part in the study by signing an informed consent form; Mini-Mental State Examination (MMSE)^5^ score above 17.

### Procedures

The experimental group participated in the intervention on twice weekly basis for three consecutive months, from 9:00AM to 3:30PM. The activities available to AD patients were: cognitive rehabilitation, computerized cognitive stimulation, speech therapy, occupational therapy, art therapy, physical training, physiotherapy, and cognitive stimulation through reading and logic games. Each activity was offered once a week, and undertaken in group sessions of one and a half hours. A psychological support group and psycho-educational workshops were available to patients’ family members and caregivers twice a week, from 10:00AM to 11:30AM. The purpose of these meetings was to explain the clinical course of the disease and its implications, as well as to encourage the exchange of personal accounts and experiences among group participants.

The control group of AD patients, which was not subject to any intervention, attended the initial evaluation and a second evaluation after a three-month interval, where those who showed interest could participate in the above-mentioned activities after the research protocol was completed.

### Materials

All patients and family members or caregivers were evaluated at the beginning and upon completion of the program by an interviewer who was blinded to group assignment. The MMSE (Mini-Mental State Examination)^5^ and GDS (Geriatric Depression Scale)^6^ were applied to assess the cognitive parameters and the presence of depression symptoms. Quality of life was assessed through the Quality of Life in AD evaluation scale (QOL-AD),^7^ translated, adapted and validated for use in Brazil by Novelli.^8,9^ The Brazilian version of the QdV-DA was used with patients, as well as with their family members or caregivers, to obtain their perceptions regarding the quality of life of the patient in their care. Interviewing patients and family members or caregivers about patients’ QOL is relevant because they may have different perceptions of this construct.

In order to assess qualitative and subjective aspects of the QOL concept, the patients in the experimental group only were asked an open question before and after the program. The question was: “How is your quality of life? Please comment.” This question was chosen in order to restrict possible biases.

### Qualitative analyses of the open question

The content of the answers to the open question was examined using the Collective Subject Discourse (CSD)^10^ methodology. This methodology comprises a set of procedures for tabulating and organizing the discourse data arising from the different accounts given by study participants. The CSD is the sum of individual discourses and therefore expresses the collective views of a group.

According to Teixeira and Lefevre,^11^ CSD is a methodological tool intended for making social representations more meaningful and explicit, thus enabling one to view a certain social group – in this case, elderly patients with AD – as the author and producer of a common discourse shared by its members. CSD thus aims to rebuild – from pieces of individual discourse – as many summarized discourses as deemed necessary to express a given thought or a social representation of a phenomenon, much like a jigsaw puzzle.

The steps of the CSD methodology are:

1) Analysis of answers separately;
2) Selection of key statements in each discourse;
3) Identification of the central idea of each key statement;
4) Grouping of central ideas with similar, equivalent or complementary meaning;
5) Building of CSD, which is the summary of a group’s fundamental ideas.

The question asked in the present study was devised to elicit the perceptions of AD patients concerning their QOL, and to investigate whether it was possible for these individuals, at their respective stage of the disease, to critically self-assess their QOL. Therefore, this served as a further efficacy parameter of the influence of a non-pharmacological intervention on patient QOL.

### Data analyses for QOL-AD quantitative variables

Kolmogorov-Smirnov tests were carried out to assess the normal distribution of demographic, cognitive and QOL-AD variables. All variables presented normal distribution so parametric tests were used. Student’s t test was used to compare experimental and control groups for demographic and cognitive variables. To evaluate the intervention impact on QOL, Student’s paired samples test was used to compare pre to post-test QOL scores for each group separately. In addition, delta scores were calculated (post-test score minus pre-test score) for the QOL-AD variables, and experimental and control group deltas were compared using Student’s *t* test.

## Results

The clinical and socio-demographic characteristics of the groups before intervention are shown in Table 1. The experimental group comprised twelve individuals – four men (33%) and eight women (67%) – whose mean age was 77.0 (±5.8 years). Eight patients were married (67%), three were widowers (25%) and one was divorced (8%). The control group comprised seven individuals - three men (43%) and four women (57%) – whose mean age was 74.3 (±4.4 years). Three patients were married (43%) and four were widowers (57%). There was no significant difference between the experimental and the control group with regard to age, years of schooling, MMSE or GDS scores (p>0.05).

**Table 1:** Clinical and socio-demographic characteristics of the groups prior to intervention. Sao Paulo, 2008.

Variables	Experimental group (n=12)	Control group (n=7)	p value
Age (years)	77.0 (±5.8)	74.3 (±4.4)	0.30
Schooling (years)	7.6 (±4.3)	9.9 (±5.9)	0.34
MMSE	20.6 (±3.9)	23.9 (±3.6)	0.08
GDS	4.6 (±3.0)	5.3 (±4.3)	0.68

Student's t test. Values correspond to mean ± standard deviation.

The results from the MMSE, GDS, and the QOL-AD scale, administered to the patients and their respective family members, with regard to their perceptions of the quality of life of the patient at the beginning and end of the study are depicted in Table 2. The delta values for these variables are also shown in Table 2.

**Table 2:** Scales and test results before and after psychosocial intervention. São Paulo, 2008.

Group	Before intervention	After intervention	Delta	p value
MMSE				
Experimental	20.6 (3.9)	18.8 (4.7)	-1.8	0.79
Control	23.9 (3.6)	22.6 (3.3)	-1.3	
GDS				
Experimental	4.6 (3.0)	3.6 (3.0)	-1.0	0.52
Control	5.3 (4.3)	5.3 (4.7)	0.0	
QOL-AD Patient				
Experimental	35.8 (5.3)	36.5 (6.2)	0.7	0.23
Control	35.6 (7.7)	33.9 (8.4)	-1.7	
QOL-AD Family Member/ Caregiver				
Experimental	30.3 (6.5)	31.0 (7.0)	0.7	0.47
Control	30.9 (6.8)	34.1 (6.9)	3.2	

p value refers to comparison between delta values for control and experimental groups using Student's t test.

The groups were compared before and after the intervention by means of Student’s *t* test for independent samples. No statistically significant difference between the groups was noted considering the two test applications. In addition, comparisons were made between the pre and post intervention performances for each separate group using Student’s *t* test for paired samples. This analysis showed no significant change from pre to post-test, except among the control group on the MMSE. In this case, a significant post-test decrease was detected.

Comparison of the deltas for the two groups revealed no statistically significant difference. This result suggests that the post-intervention gain did not differ from one group to the other.

Concerning the CSD analysis, the question “How is your quality of life? Please comment.” was answered in writing by the patient on a blank sheet of paper and the average duration of this activity was eight minutes. The data presented below refers to the experimental group prior to and after intervention. Table 3 highlights the central ideas and the corresponding discourse expressed during the interview of the experimental group in the pre-intervention phase.

**Table 3:** Central Idea and Collective Subject Discourse of 12 patients with mild to moderate AD in answering the question: "How is your quality of life? Please comment." (Pre-intervention). Sao Paulo, 2008.

**Central Idea (1) - 5 individuals**	**Collective Subject Discourse (1)**
Quality of Life is excellent	My quality of life is excellent, I have a wonderful family and I am well nourished, I have nothing to complain about, praise the Lord.
	
**Central Idea (2) - 4 individuals**	**Collective Subject Discourse (2)**
Quality of Life is good	I think my quality of life is quite good, it is better than it was before, but I still forget things.
	
**Central Idea (3) - 2 individuals**	**Collective Subject Discourse (3)**
Quality of Life is reasonable	My quality of life is average because I don't feel fulfilled; there is a lack of conversation with my partner.
	
**Central Idea (4) - 1 individual**	**Collective Subject Discourse (4)**
Quality of Life is terrible	My quality of life is terrible because I have to always rely on the help of others. I never go out alone; I only go out when escorted.

Overall, the discourse of the twelve patients was poor, considering only a few comments were added in answering the question (13.6 words on average per participant). Four central ideas were recorded, namely, QOL is excellent, good, reasonable and terrible.

Table 4 highlights the central ideas and the corresponding discourse from the interview of the experimental group after intervention.

**Table 4:** Central Idea and Collective Subject Discourse of 12 patients with mild to moderate AD in answering the question: "How is your quality of life? Please comment." (Post intervention). Sao Paulo, 2008.

**Central Idea (1) - 5 individuals**	**Collective Subject Discourse (1)**
Quality of Life is excellent	My quality of life is excellent, always improving.
	
**Central Idea (2) - 3 individuals**	**Collective Subject Discourse (2)**
Quality of Life is good	From my standpoint, my quality of life is quite good.
	
**Central Idea (3) - 1 individual**	**Collective Subject Discourse (3)**
Quality of Life is reasonable	My quality of life is average, completely calm.
	
**Central Idea (4) - 1 individual**	**Collective Subject Discourse (4)**
Quality of Life is not good	My quality of life is not that good because I feel worthless and can't do things. I feel good for nothing, I liked attending my classes and taking care of my affairs so much, and now I can't, I'm too old.
	
**Central Idea (5) - 6 individuals**	**Collective Subject Discourse (5)**
Quality of Life has changed	Believe it or not, my quality of life has radically changed. At my age, too, that's why I was surprised. After attending the university's workshops I feel much better. I used to forget things all the time and found this terrible. Now I am happier, I feel better about my mind, I take the medicine and feel better in general, even my children have noticed. I am very pleased with the instructors, who are young and wonderful. I am very glad, and always eager to start our day of activities. I am sure I get better every day. I've been treated with medicine and I've made many friends. I like the classes here, I participate in the activities, and in this fashion time goes by while we amuse ourselves. I thank all of the instructors.

It was noteworthy that the participants wrote more about their QOL after the intervention (48.5 words per participant on average). Five central ideas were established: QOL is excellent, good, average, poor, and QOL changed. The central ideas for excellent and good QOL appeared in analyses both prior to and after intervention. In the third central idea, QOL is reported as being average and completely calm, indicating that the variable QOL can be interpreted in different ways. In the fourth central idea, “Quality of life is not good”, the CSD indicates patients’ dissatisfaction over their situation. In the fifth central idea, present in the accounts of half the individuals undergoing intervention – “Quality of life has changed”, the CSD indicates that the QOL of AD sufferers was positively influenced by the psychosocial intervention.

## Discussion

The present study sought to investigate the effectiveness of a multi-professional intervention of 24 sessions based on the view of the AD patient and that of their family member or caregiver regarding the patient’s QOL. The results from the QOL-AD scale showed no significant differences between the experimental and the control groups after participating in the program in terms of the perception of patients and their caregivers. However, the qualitative analysis of the patients’ speech at the beginning and end of the program using the CSD^10^ methodology indicated positive changes in QOL perception after the intervention, especially in the accounts of six patients classified as “QOL has changed”.

A decrease in MMSE performance in both the control group – which underwent no psychosocial intervention – and the experimental group was observed. This is in keeping with the progressive nature of AD, and this information suggests that the intervention did not succeed in stabilizing the decline in cognitive performance of the participants.

Previous Brazilian papers related to rehabilitation of AD patients^12,13^ found that a slight improvement or stabilization of cognition and behavioral symptoms occurred. Bottino and colleagues^12^ carried out a psychosocial intervention similar to that described in this paper. The six patients with mild AD who underwent weekly interventions for six months displayed stabilization or slight improvement in cognitive performance and daily activities. Stabilization or reduction of depression and anxiety levels was also observed. Abrisqueta-Gomez and colleagues^13^ demonstrated cognitive enhancement, functional stabilization and improvement of behavioral problems in three patients diagnosed with mild to moderate AD after twelve months of psychosocial intervention. However, the reported benefits were not sustained throughout the second year of intervention. For this reason, the absence of gains in clinical parameters in the present study may have been due to the relatively short duration of the intervention. An extended intervention may be needed to affect these parameters. Another possibility is that these instruments are not sufficiently sensitive to detect the changes that may have occurred.

Even though QOL is at present recognized as one of the core targets of healthcare programs,^14^ few research studies have included this variable as a measure of effectiveness in therapeutic interventions. A considerable number of papers have sought to identify which factors constitute the QOL construct and how best to measure them. However, moves to use QOL as an outcome measure of interventions remains incipient.

Aisen and colleagues,^15^ for instance, conducted a random, double-blind study which aimed to determine whether treatment with anti-inflammatory drugs (Naproxen and Rofecoxib) would lessen cognitive decline in AD patients. A total of 351 participants with mild to moderate AD were recruited, and subsequently divided into three groups: placebo, Naproxen and Rofecoxib. The QOL-AD was also used. The three groups showed a decline in QOL after the treatment, an observation suggesting that QOL is a phenomenon connected to aspects of life not necessarily addressed during pharmacological intervention.

Spector and colleagues^16^ carried out a cognitive intervention program in 201 AD patients. The program lasted for seven weeks, and involved 45-minute sessions run twice a week (14 sessions). The measured parameters included cognition, quality of life, communication, behavior, global functioning, depression and anxiety. Participants were split into a control group (N=115) and an experimental group (N=86). By the end of the intervention a significant improvement in QOL-AD and cognition was observed in the experimental group. Use of a larger sample and increased statistical power to detect changes may explain the difference between the present results and the study of Spector and colleagues.

Orrell and Spector^17^ assessed the effectiveness of a 16-week maintenance program following the cognitive stimulation carried out by Spector^16^ and colleagues in 2003. The group that participated in the maintenance program displayed an improvement in cognitive functioning and an increase in MMSE score. Stabilization of QOL measured by the QOL-AD scale was recorded. The group not undergoing maintenance therapy demonstrated poorer QOL.

Nevertheless, another study by Longsdon and colleagues found improvement in QOL for patients with AD^18^. A total of 95 AD patients participated in a 12-week intervention. These interventions adopted strategies seeking to lessen depression and anxiety symptoms in patients and caregivers. The QOL-AD was included in the protocol. Depression symptoms of caregivers were shown to be reduced. The reduction of depression and anxiety symptoms in patients was associated with improvement in patients’ QOL reported by their caregivers and with improvement in the QOL of the actual caregiver.

Overall it should be noted that research on QOL is incipient and uses different methodological strategies. Nevertheless, according to Brodaty^19^ who conducted a meta-analysis study, it is possible to assert that psychosocial interventions potentially benefit patients and their family in spite of methodological differences.

In the present paper, no significant statistical difference was observed between groups regarding QOL measured by the QOL-AD. This outcome may be a consequence of the small sample size in comparison with the research carried out by Longsdon and colleagues^18^ in 2005. Alternatively, the outcome could have resulted from the shorter intervention period vis-à-vis Orrell and Spector’s^17^ study in 2005, or may have stemmed from the fact that QOL is a multidimensional phenomenon influenced by a variety of factors which were not controlled in this study, such as loss of friends or family over the years. It is also possible that the QOL-AD scale has been incapable of detecting subtle changes in the QOL of participants. Quantitative scales may be unable to fully address QOL-related questions, especially in small groups. The CSD methodology enabled detection of changes in patients’ perceptions regarding their QOL.

Under one of the central ideas from the patients’ discourse before intervention – “Quality of Life is good” – patients stated that their QOL was good although they noticed they forgot things. This is evidence that individuals suffering from mild to moderate AD remain discerning and exercise sound judgment regarding their present situation. This information is in line with the views of Logsdon,^7,20^ who claimed that patients with a an MMSE score of 18 or higher are capable of critically assessing their QOL, as well as being able to participate in the framing of psychometric tools by giving their opinion on the domains used in assessments.

Under another central idea before intervention – “Quality of Life is terrible” – one patient declared that their QOL was terrible because they depended on the assistance of someone else. Being ill is associated with the limitations imposed by the illness and with the way the patient deals with their condition.

It is noteworthy that after the intervention, the average number of words used by patients to express themselves spontaneously increased from 13.6 to 48.5. Half the patients declared that their “Quality of Life has changed”, while CSD results suggested that the psychosocial intervention positively affected the QOL of patients by expanding their friendship networks and increasing their motivation, besides the pleasure of attending the program. Finally, participants provided vital reports to further the discussion and analysis of the QOL variable, which in turn is now one of the core objectives of healthcare.

A few methodological limitations in the present research should be mentioned, namely the small sample size and short duration of the intervention – three months – compared to the parameters of previous studies (Abrizqueta-Gomes, 2004). In addition, control group participants were not asked the open question about QOL. Control group answers at post-test could potentially have been used as a comparison parameter. Nevertheless, the results from this study suggest that QOL may change after intervention. Despite the fact that the QOL-AD scale detected no significant change in QOL, the CSD methodology enabled detec tion of changes in patients’ perceptions regarding QOL. Therefore, qualitative data suggests that combining drug treatment with psychosocial intervention may prove to be an effective strategy to enhance the QOL of AD sufferers.
